# Design, Fabrication, and Testing of a Bulk Micromachined Inertial Measurement Unit

**DOI:** 10.3390/s100403835

**Published:** 2010-04-14

**Authors:** Honglong Chang, Qiang Shen, Zhiguang Zhou, Jianbing Xie, Qinghua Jiang, Weizheng Yuan

**Affiliations:** Micro and Nano Electromechanical System Laboratory, Northwestern Polytechnical University, 127#, Youyi West Road, Xi’an, Shaanxi, China; E-Mail: yuanwz@nwpu.edu.cn (W.-Z.Y.)

**Keywords:** MEMS, MIMU, bulk micromachining, gyroscope, accelerometer

## Abstract

A bulk micromachined inertial measurement unit (MIMU) is presented in this paper. Three single-axis accelerometers and three single-axis gyroscopes were simultaneously fabricated on a silicon wafer using a bulk micromachining process; the wafer is smaller than one square centimeter. In particular, a global area optimization method based on the relationship between the sensitivity and layout area was proposed to determine the layout configuration of the six sensors. The scale factors of the X/Y-axis accelerometer and Z-axis accelerometer are about 213.3 mV/g and 226.9 mV/g, respectively. The scale factors of the X/Y-axis gyroscope and Z-axis gyroscope are about 2.2 mV/°/s and 10.8 mV/°/s, respectively. The bias stability of the X/Y-axis gyroscope and the Z-axis gyroscope are about 2135 deg/h and 80 deg/h, respectively. Finally, the resolutions of X/Y-axis accelerometers, Z-axis accelerometers, X/Y-axis gyroscopes, and Z-axis gyroscopes are $0.0012\;g/\sqrt{\text{Hz}}$, $0.0011\;g/\sqrt{\text{Hz}}$, $0.314\;{}^{\circ}\;/s/\sqrt{\text{Hz}}$, and $0.008\;{}^{\circ}\;/s/\sqrt{\text{Hz}}$, respectively.

## 1. Introduction

Inertial navigation requires a measurement with six degrees of freedom (DOF) in three-dimensional space, namely the ability to move forward or backward, up or down, left or right (translation in three perpendicular axes) combined with rotation about three perpendicular axes (pitch, yaw, roll). A micromachined inertial measurement unit (MIMU) is used to output signals proportional to the rotation and translational motion of a carrier in six DOFs from, respectively, micromachined angular rate sensors and acceleration sensors. Traditionally, these separated inertial sensors are mechanically mounted orthogonally in one or two substrates to output the three-axis angular rate and acceleration measurement signals [1]. In this mechanical assembling process, it is difficult to achieve exact mutual orthogonality between the sensors. Orthogonality aberrations degrade the accuracy of the subsequent processes, such as attitude determination or inertial navigation. A variety of multi-axis inertial sensors, such as tri-axis micro gyroscopes [2–4] or tri-axis micro accelerometers [5–8], can alleviate this misalignment problem in MIMU. Moreover, such technology allows the MIMU to be even smaller because the multi-axis sensing only requires one inertial mass. However, the cross-axis coupling within the multi-axis sensor limits the performance of the sensor. Designing a tri-axis sensor with only one inertial mass is a challenging task and requires a creative, clever design.

Integrated MIMU (IMIMU) technology can avoid the misalignment problem without tri-axis sensors. In IMIMU, different single-axis inertial sensors are fabricated simultaneously on a single chip with a surface or bulk micromachining process. Thus, the mutual orthogonality in IMIMU is inherently guaranteed by the design. Additionally, compared with tri-axis sensors, a single-axis inertial sensor is more rugged, and easier to design. Furthermore, cross coupling within a tri-axis sensor is successfully avoided. The first IMIMU was implemented using a surface micromachining process in 1998 [9]. It integrates three accelerometers, a dual-axis gyroscope, and a Z-axis gyroscope into a 1-cm^2^ chip, including the interface circuitry. Luo Hao integrated two accelerometers and a gyroscope into a single chip using a surface process in 2003 [10]. It did not include an X/Y-axis gyroscope or a Z-axis accelerometer. Currently, surface micromachined IMIMUs have been commercialized successfully by Analog Devices, Inc (Norwood, MA, USA).

However, there is no report on bulk micromachined IMIMU so far. The major difficulty is the lack of a mature monolithic bulk micromachining process. Some monolithic bulk processes have emerged recently [11–15]. Unfortunately, they are not mature enough to realize a complex device like IMIMU. It is widely accepted that the bulk process could achieve better performance than the surface process for the inertial sensor. Today, inertial sensors with the highest accuracy [16,17] are nearly all fabricated by bulk micromachining process, where the larger proof mass can reduce mechanical noise to improve performance. Therefore, people usually utilize a two-chip integration scheme to realize bulk micromachined MIMU. In this two-chip approach, the six mechanical sensing elements are simultaneously fabricated on a chip and packaged with a circuit chip that reads out the various signals and generates the corresponding signal. Donato Cardarelli proposed a two-level conceptual MIMU design scheme based on the standard dissolved wafer process in 2002 [18]. However, no further fabrication or test results with this bulk micromachined MIMU have been reported. In our previous work [19], we established a two-chip MIMU using a bulk micromachining process. However, the yield rate of fabricating the mechanical chip is very low, which partially explains why there has not been a commercial bulk micromachined MIMU until now.

There are many difficulties when fabricating the six inertial sensors simultaneously through the bulk micromachining process. First, there is a tradeoff between area limitation and sensor performance. For a single inertial sensor, greater area usually means better performance because the performance is usually proportional to the inertial mass. However, greater area reduces the accuracy of micromachining process. In our bulk process, the minimum line width is 2 μm when the layout area is less than 8 mm × 8 mm. However, the minimum line width will become 10 μm when the layout area is larger than 10 mm × 10 mm. Therefore, a trade-off must be made to optimize a total of six sensors in a limited area. Secondly, the reactive ion etching (RIE) lag effect [20,21], which does not appear as a major problem in the surface micromachining, and notching effect [22] in deep RIE (DRIE) are challenges when generating a bulk micromachined MIMU. The six sensors inevitably have different line widths. Consequently, different etching depths caused by the RIE lag effect definitely hurt the performance of inertial sensors. Finally, the residual stress caused by the anodic bonding and chemical mechanical planarization (CMP) in the bulk process [23–24] cause a large mode mismatch for gyroscopes, which is a fatal bug for gyroscopes. In this paper, we present solutions to these problems to establish a bulk micromachined two-chip MIMU based on our previous work.

## 2. Topologies Selection for the Inertial Sensors Based on a Two-Structure-Layer Bulk Process

**Figure 1.**
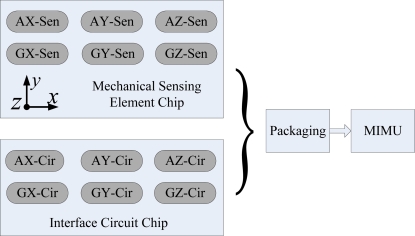
Schematic of the two-chip integration scheme.

The proposed MIMU integrates two chips, as shown in Figure 1, where the letters A and G represent the accelerometer and gyroscope, respectively. The mechanical sensing element chip consists of sensing structures for six single-axis inertial sensors, *i.e.*, three accelerometers and three gyroscopes. While the interface circuit chip consists of the six interface circuits that match the corresponding sensors. The two chips are packaged together to form the final MIMU device.

**Figure 2.**
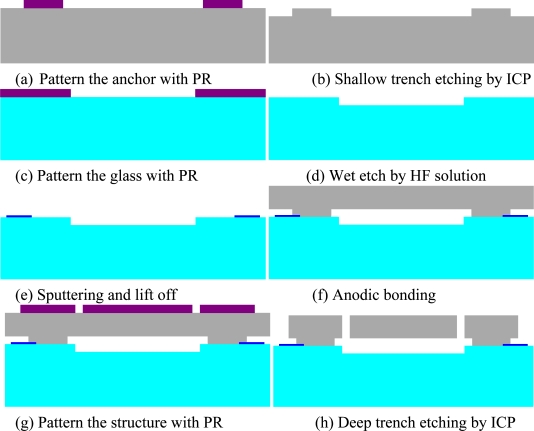
The process flow for the mechanical sensing element chip.

The first step when designing the bulk micromachined MIMU is to determine its topology for each single-axis inertial sensor based on a bulk process. The sandwich process or other processes with more than three structure layers are very complex and greatly reduce the yield rate to MIMU. A two-structure-layer process, as shown in Figure 2, is used to design the MIMU. Fabrication begins with a P-type single-crystalline silicon wafer. First, the photo resist (PR) is coated on the wafer to form the mask of anchors, as shown in step (a). In step (b), inductively coupled plasma (ICP) etching is used to etch shallow trenches to form anchors, and in step (c), PR is coated on the glass wafer. In step (d), a deep trench is wet etched on the glass wafer to increase the distance between the proof mass and the substrate. In step (e), a layer of metal is sputtered on the glass to form electrodes. In step (f), anodic bonding is used to bond the glass and silicon wafer together. At this step chemical mechanical polishing is used to reduce the thickness of the silicon wafer to meet the actual demand. In step (g), PR is coated on the back side of the silicon wafer. Finally, in step (h), ICP is used again to form moving structures, such as comb drives or damping holes.

It is obvious that the major process flow is similar to our previous one [19]. However, some improvements have been proposed to ensure an acceptable MIMU yield rate. The thickness of the proof mass was reduced to 65 microns from 80 microns. One additional mask was provided to add a trench in the glass to increase the distance between the proof mass and the substrate for some sensors, including the Z-axis gyroscope and X/Y-axis accelerometer. Thus, the slide film damping in these devices is greatly reduced to improve the quality factors for Z-axis gyroscopes because the MIMU is packaged at one atmosphere.

**Figure 3.**
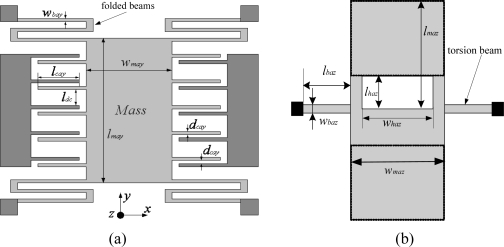
(a) The topology of the X/Y-axis accelerometer. (b) The topology of the Z-axis accelerometer.

In this study, we use the most typical topologies for inertial sensors instead of novel designs to ensure a better yield rate because they have been verified in many ways. There are many typical topologies for accelerometers [25–30]. Most lateral accelerometers fabricated by surface or bulk micromachining processes can be used as the X/Y-axis accelerometer in the proposed MIMU. The vertical accelerometers, which require the sandwich process, cannot be used as the Z-axis accelerometer in the proposed MIMU. Finally the topologies of the accelerometers are chosen as shown in Figure 3. After experimental verification with the previous MIMU, the topologies are proved to be effective and are still used in this version of MIMU.

**Figure 4.**
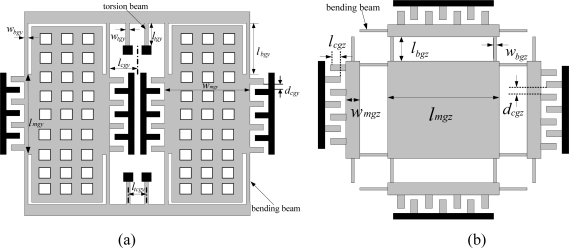
(a) X/Y-axis gyroscope topology in the two-chip MIMU. (b) Z-axis gyroscope topology in the two-chip MIMU.

There are also many typical topologies for gyroscopes [31–36] that are compatible with the above-mentioned process flow. In the current version, there are many essential improvements on gyroscopes to increase the yield rate of the MIMU. The vibrating wheel gyroscope in the previous version was difficult to fabricate. Thus, we used the tuning fork gyroscope instead [31]. The Z-axis gyroscope with interdigitated electrodes in the sense mode [32] was difficult to fabricate because the large metal electrodes in the glass wafer are hard to remove. Therefore, we used a symmetrical Z-axis gyroscope topology [33] to replace it. Finally, the topologies of the gyroscopes are chosen as shown in Figure 4.

## 3. Layout Optimization for the Mechanical Sensing Elements

After choosing a feasible topology for each inertial sensor, we must determine the dimensions for each sensor. However, the MIMU consists of six separated inertial sensors, which means the usual device optimization approaches, such as a genetic algorithm (GA) [36] or simulated annealing algorithm (SAA) [37], are not suitable to be applied directly to MIMU design. Therefore, area occupations for each sensor should be determined first before the detailed design for the single sensor. In this paper, we propose an area optimization approach to establish the layout configuration for MIMU.

### 3.1. Common Geometry Patterns for MIMU

To alleviate the RIE lag effect in the bulk micromachining process, the geometry patterns for the six sensors should be set as close as possible. As a result, the inertial masses, the suspension beams, the damping holes, and the comb capacitors should be given a very similar geometry pattern with very close dimensions, which we call *common geometry patterns*.

Some common geometry patterns are simply determined by the process rules. For example, the gap and the width of comb fingers in comb drives or comb capacitors are both set as 4 microns to obtain the maximum capacitance in a limited area. In addition to these two parameters, we also set the overlap and the length of comb fingers in an identical configuration as shown in Table 1.

Some common patterns, such as resonant frequency and inertial mass, are mainly determined by the requirements of the sensor. It is important to choose a common resonant frequency for these inertial sensors according to the requirements because the sensitivity depends on it. In this paper, 4,000 Hz is selected initially for these sensors to ensure both a large sensitivity and adequate resistance to disturbances. The area of the inertial mass is also an important parameter because it is related to either the initial capacitance or capacitance variance. It is mainly determined by the capacitance detection capability. Consequently, the suspension beams for these inertial sensors also have a common configuration initially. Two kinds of beams largely determine the spring constant of the corresponding working modes: bending beams and torsional beams, which are used to determine the spring constant of the Z-axis accelerometer and the sense mode of X/Y-axis gyroscope. We provide a list of initial empirical values for these suspension beams in Table 1. These common geometry patterns not only alleviate the RIE lag effect but also simplify the deduction of the relationship between sensitivity and layout area.

### 3.2. Sensitivity *versus* Layout Area

Because there is an area limitation to design inertial sensors using the bulk micromachining process, it is useful to find the relationship between the performance and the area. We deduce a simplified relationship between the sensitivity and the layout area with some assumptions and some available dimensions.

The sensitivity and resonant frequency of the X/Y-axis accelerometer can be described as the following:

(1) $$S_{\mathrm{ay}}=\frac{\Delta c}{a}=\frac{1}{\omega_{\mathrm{ay}}{}^{2}}n\frac{\varepsilon_{0}l_{\mathrm{cay}}h}{d_{\mathrm{cay}}{}^{2}}$$

(2) $$\omega_{\mathrm{ay}}=\sqrt{\frac{4Ew_{\mathrm{bay}}{}^{3}}{\rho w_{\mathrm{may}}l_{\mathrm{may}}l_{\mathrm{bay}}{}^{3}}}$$

where *l_may_*, *w_may_*, *l_bay_*, *w_bay_*, *l_cay_*, *d_cay_* are shown in Figure 3 (a); *l_may_* and *w_may_* are the length and width of the inertial mass of the X/Y-axis accelerometer; *l_bay_* and *w_bay_* are the length and width of the bending beams; *l_cay_* is the overlap of the comb fingers; *d_cay_* is the gap between capacitor plate; *n* is the number of comb fingers; *h* is the thickness of the structure; *ω_ay_* is the natural resonant frequency of the X/Y-axis accelerometer; *ε_0_* is the dielectric constant of the air; *E* is Young’s modulus of silicon; and *ρ* is density of silicon.

It can be seen from Equation (1) that the sensitivity of the X/Y-axis accelerometer is proportional to the number of comb fingers, which is evident in some parameters, such as the overlap, thickness and gap, which are shown in Table 1. As for the biased comb capacitors, *l_dc_* is usually set to be much bigger than the gap *d_cay_*; here, *l_dc_* = *d_cay_*. To ensure the inertial mass of the accelerometer is large enough, we assumed that the area occupied by the biased comb capacitors is about a quarter of the total area of the accelerometer. Therefore, we can assume that the sensitivity of the X/Y-axis accelerometer is only related to its area. To further simplify the relationship, we assumed that the area of the X/Y-axis accelerometer is represented by an equivalent square with an equivalent side length. Consequently, the equivalent side length for X/Y-axis accelerometer is approximated as $L_{\mathrm{ay}}=\sqrt{2\times n\times l_{\mathrm{cay}}\times d_{\mathrm{cay}}\times 4\times 4}$ when the area occupied by the fingers is ignored.

As for the Z-axis accelerometer, the sensitivity and resonant frequency formulas are:

(3) $$S_{\mathrm{za}}=\frac{\Delta C}{a}=\frac{3}{2\omega_{\mathrm{za}}{}^{2}}\frac{\frac{w_{\mathrm{haz}}}{w_{\mathrm{maz}}}{\left(\frac{l_{\mathrm{haz}}}{l_{\mathrm{maz}}}\right)}^{2}}{\left\{2-{\left(\frac{l_{\mathrm{haz}}}{l_{\mathrm{maz}}}\right)}^{3}+\left(1-\frac{w_{\mathrm{haz}}}{w_{\mathrm{maz}}}\right){\left(\frac{l_{\mathrm{haz}}}{l_{\mathrm{maz}}}\right)}^{3}\right\}}\frac{\varepsilon l_{\mathrm{maz}}w_{\mathrm{maz}}\left(1-{\left(\frac{l_{\mathrm{haz}}}{l_{\mathrm{maz}}}\right)}^{2}\right)}{d_{0}^{2}}$$

(4) $$\omega_{\mathrm{za}}=\sqrt{\frac{6G\beta w_{\mathrm{baz}}{}^{3}}{\rho l_{\mathrm{baz}}w_{\mathrm{maz}}l_{\mathrm{maz}}{}^{3}\left(2-{\left(\frac{l_{\mathrm{haz}}}{l_{\mathrm{maz}}}\right)}^{3}+\left(1-\frac{w_{\mathrm{haz}}}{w_{\mathrm{maz}}}\right){\left(\frac{l_{\mathrm{haz}}}{l_{\mathrm{maz}}}\right)}^{3}\right)}}$$

where *l_baz_*, *w_baz_*, *l_maz_* ,*w_maz_*, *l_haz_* ,*w_haz_* are shown in Figure 3 (b); *l_baz_* and *w_baz_* are the length and width of the bending beams, respectively; *l_maz_* and *w_maz_* are the length and width of the inertial mass, respectively; *l_haz_* and *w_haz_* are the length and width of the hole, respectively; *d*_0_ is the gap between structure and the substrate; *ω_za_* is the natural resonant frequency of the Z-axis accelerometer; *G* is torsion modulus; and *β* is torsion coefficient.

It can be seen from Equation (3) that the sensitivity of the Z-axis accelerometer is related to the area of the inertial mass (*l_maz_* × *w_maz_*) and the ratio *l_haz_*/*l_maz_*. The sensitivity reaches its maximum when the ratio is about 0.7. Furthermore, in order to ensure a large stiffness in the Z-axis direction, we set *w_haz_* = 0.5 × *w_maz_*. Therefore, we can obtain an equivalent side length for the Z-axis accelerometer, $L_{\mathrm{az}}=\sqrt{{2w}_{\mathrm{maz}}\times l_{\mathrm{maz}}}$, with a similar assumption.

For the X/Y-axis gyroscope, the sensitivity and resonant frequency formulas are:

(5) $$S_{\mathrm{gy}}=4\times {\left(\frac{39}{64}\right)}^{2}\frac{\varepsilon^{2}\rho h^{2}l_{\mathrm{mgy}}w_{\mathrm{mgy}}{}^{2}{\left(l_{\mathrm{mgy}}+2l_{\mathrm{bgy}}\right)}^{2}}{\omega_{\mathrm{dgy}}\left(\mu^{*}l_{\mathrm{cgy}}hl_{\mathrm{mgy}}d_{0}{}^{2}+\mu^{*}w_{\mathrm{mgy}}\left(l_{\mathrm{mgy}}+2l_{\mathrm{bgy}}\right)d_{\mathrm{cgy}}{}^{2}d_{0}\right)c_{s}}V_{p}v_{a}$$

(6) $$\omega_{\mathrm{dgy}}=\sqrt{\frac{256Ew_{\mathrm{bgy}}{}^{3}}{39\rho \left(l_{\mathrm{mgy}}+2l_{\mathrm{bgy}}\right)w_{\mathrm{mgy}}l_{\mathrm{bgy}}{}^{3}}}$$

(7) $$\omega_{\mathrm{sgy}}=\sqrt{\frac{128G\beta w_{\mathrm{tgy}}{}^{3}}{39\rho w_{\mathrm{mgy}}l_{\mathrm{tgy}}(l_{\mathrm{mgy}}+2l_{\mathrm{bgy}}){\left(\frac{w_{\mathrm{mgy}}}{2}+l_{\mathrm{cgy}}\right)}^{2}}}$$

where *l_mgy_*, *w_mgy_*, *l_bgy_*, *w_bgy_*, *l_tgy_*, *w_tgy_*, *l_cgy_* are shown in Figure 4 (a); *l_tgy_* and *w_tgy_* are the length and width of the torsion beams, respectively; *d_cgy_* is the width of the comb fingers; *d*_0_ is the gap between structure and substrate; *h* is thickness of the structure; *ω_dgy_* is the natural resonant frequency of drive mode; *ω_sgy_* is natural resonant frequency of sense mode; *ρ* is the density of the silicon; μ* is the dynamic viscosity of the air; *V_p_* is DC voltage; and *v_a_* is the AC voltage.

It can be seen from Equation (5) that the sensitivity of the X/Y-axis gyroscope is related to its area of inertial mass because the beam dimensions of the beams were given a group of initial values. An equivalent side length for the X/Y-axis gyroscope can be given as $L_{\mathrm{gy}}=\sqrt{2\times \left(w_{\mathrm{mgy}}+l_{\mathrm{cgy}}\right)\left(l_{\mathrm{mgy}}+2l_{\mathrm{bgy}}\right)}$.

As for the Z-axis gyroscope, the sensitivity and resonant frequency formulas are :

(8) $$S_{\mathrm{gz}}=2\rho \frac{\varepsilon_{0}{}^{2}}{\omega_{\mathrm{gz}}}\frac{h^{3}}{{\left(\frac{\mu^{*}l_{\mathrm{cgz}}h}{l_{\mathrm{mgz}}}+\frac{\mu^{*}d_{\mathrm{cgz}}{}^{2}}{d_{0}}\right)}^{2}}V_{p}v_{a}$$

(9) $$\omega_{\mathrm{gz}}=\sqrt{\frac{8Ew_{\mathrm{bgz}}{}^{3}}{\rho l_{\mathrm{mgz}}{}^{2}l_{\mathrm{bgz}}{}^{3}}}$$

where *l_mgz_*, *w_mgz_*, *l_bgz_*, *w_bgz_* are shown in Figure 4 (b); *d_cgz_* is the width of the comb fingers; *h* is thickness of the structure; *ω_gz_* is the natural resonant frequency of the Z-axis gyroscope; and *V_p_* is DC voltage; *v_a_* is AC voltage.

It can be seen from Equation (8) that the sensitivity of the Z-axis gyroscope is related to its area of inertial mass because the beam dimensions were given a group of initial values. An equivalent side length for Z-axis gyroscope can be given as *L_gz_* = *l_mgz_* + 2 × (*l_bgz_* − 50).

All the formulas for the equivalent side length are listed in Table 2. Based on these formulas, the sensitivity is dependent on just one variable related to its area. We can use this relationship in our global optimization to determine the layout configuration for these six sensors.

### 3.3. Global Optimization Process for MIMU

**Figure 5.**
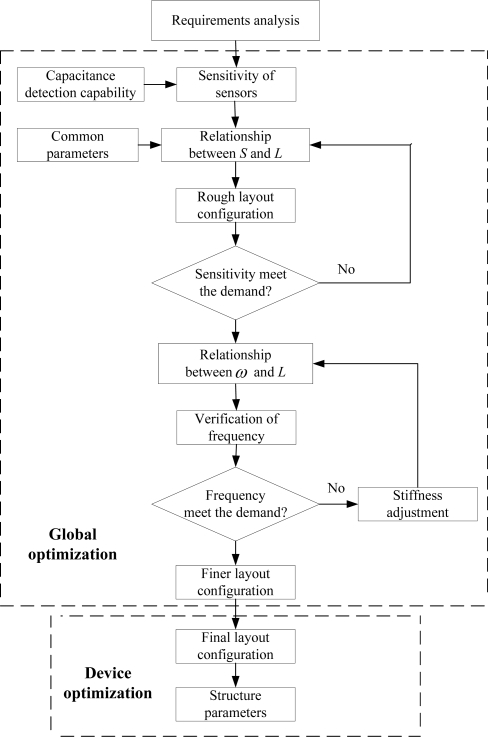
The flow of the proposed global optimization approach.

A global optimization approach is proposed to determine the layout configuration for inertial sensors in MIMU, as shown in Figure 5. The optimization begins with the requirements analysis. The requirements usually reflect the sensitivity when considering the capacitance detection capability. Then, we can use the relationship between the sensitivity and the equivalent side length to get a rough layout. The layout configuration must be adjusted further according to the relationship between the resonant frequency and the area. Then, we can get a finer layout configuration for these six inertial sensors. Given the layout configuration obtained in the global optimization stage, we can design each single sensor carefully, as usual.

**Figure 6.**
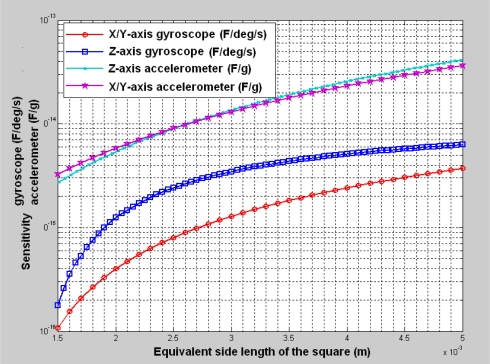
The sensitivity *versus* layout area; the natural frequency of each inertial sensor is chosen as 4,000 Hz.

**Figure 7.**
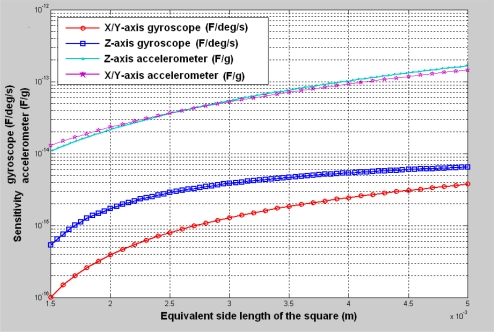
The sensitivity *versus* layout area; the natural frequency of the accelerometers is 2,000 Hz, and the natural frequency of the gyroscopes is 4,000 Hz.

As for our design case, the accuracy of the accelerometers and gyroscopes are expected to reach a navigation level [38], in which the resolution of accelerometers is about 10^−6^ g and the resolution of gyroscopes is about 10^−4^ °/s. If the detection capability of the capacitive interface circuit can reach 10^−19^ *F*, then the sensitivity of gyroscopes should be larger than 10^−15^ F/^o^/s and the sensitivity of the accelerometers should be larger than 10^−14^ F/g. Before the optimization, the natural resonant frequency of each inertial sensor was chosen as 4,000 Hz. Using the relationship between the sensitivity and equivalent side length, as plotted in Figure 6, we obtain a rough layout configuration in which the equivalent side length was determined to be 3,000–3,500 μm. This area satisfies the sensitivity requirements for both gyroscopes and accelerometers. However it is difficult to assign six sensors with a side length of about 3,500 μm within a 10,000 μm × 10,000 μm square. Therefore, we reduced the area of the accelerometers. To make the accelerometers meet the sensitivity requirements, the natural resonant frequency of the accelerometers must be reduced. Therefore, we generated a new layout configuration based on Figure 7, in which the equivalent side length of accelerometers was about 1,500–2,000 μm. Some available material or process parameters used in the optimization are listed in Table 3.

**Figure 8.**
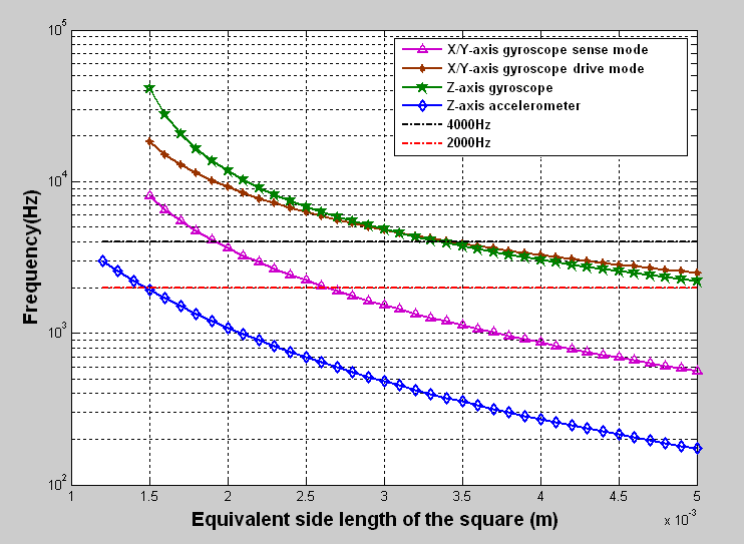
The natural resonant frequencies of the inertial sensors *versus* the layout area.

After getting the rough layout configuration, we need to prove that the natural resonant frequencies meet the requirements using the equivalent side length in the rough layout configuration. The actual natural resonant frequency of the inertial sensor *versus* the layout area is plotted in Figure 8. It is obvious that the frequency of the Z-axis gyroscope is 4,000 Hz when the equivalent side length is about 3,000 μm. However the frequency of the sense mode for the X/Y-axis gyroscope is not more than 2,000 Hz. We simply increased the frequency to 4,000 Hz by reducing the length of the torsional beams. The Z-axis accelerometer meets the frequency requirements for the given area. Therefore, we set the equivalent side length of the Z-axis accelerometer to be about 1,500 μm. The X/Y-axis accelerometer took the folded beams. Therefore, the stiffness adjustment could be made easily to meet the frequency requirements without considering the equivalent side length. Thus, we do not plot the X/Y-axis accelerometer here.

### 3.4. Device Optimization Results for MIMU

**Figure 9.**
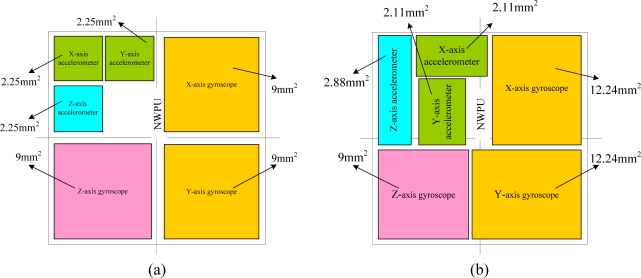
Layout configuration of the mechanical sensing element chip. (a) The layout configuration after global optimization. (b) The final layout configuration after device optimization.

After obtaining the global layout configuration for the six inertial sensors, the various device optimization approaches can be used to determine the final layout of MIMU within the given area. Because the design in this stage is very common, we skip the details and give the final dimensions of MIMU in Tables 4–7. We obtained the final layout configuration of the mechanical chip as shown in Figure 9 and Table 8.

## 4. Interface Circuit Design for Inertial Sensors

**Figure 10.**
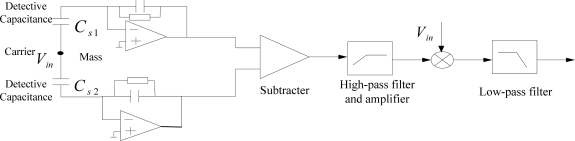
The interface circuit block diagram for the accelerometers.

**Figure 11.**
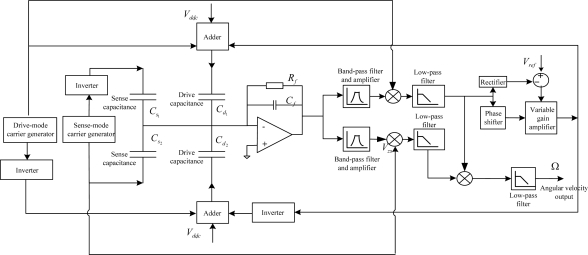
The interface circuit block diagram for the gyroscopes.

Various interface circuit design schemes exist. Here, we took some typical ones for accelerometers (Figure 10) and gyroscopes (Figure 11) in the MIMU. However, there are six different single-axis sensors in MIMU; the various signals will experience crosstalk. Therefore, special design in the interface circuit chip is needed to suppress the crosstalk. In addition to the necessary shielding, the proper choice of interface circuit frequency for the different sensors is also very helpful in suppressing crosstalk. Usually the noises of the operational amplifiers decrease with a higher frequency. Thus, we set the frequency of the interface circuit for gyroscopes to be higher than that of the accelerometers because the gyroscope is less sensitive than the accelerometer. Such a configuration is very helpful in reducing noise in the circuit.

**Figure 12.**
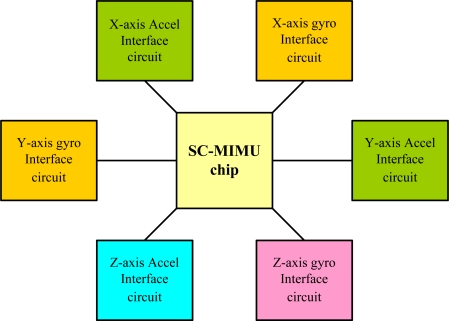
Layout configuration of the interface circuit chip in MIMU.

As in the mechanical chip, a proper layout in the circuit chip can suppress crosstalk. The circuits for the gyroscopes were placed next to the circuits for the accelerometers. Furthermore, the circuits with similar frequencies were separated. The final layout configuration of the interface circuit chip is shown in Figure 12.

## 5. Fabrication and Test of the Bulk-Micromachined MIMU

**Figure 13.**
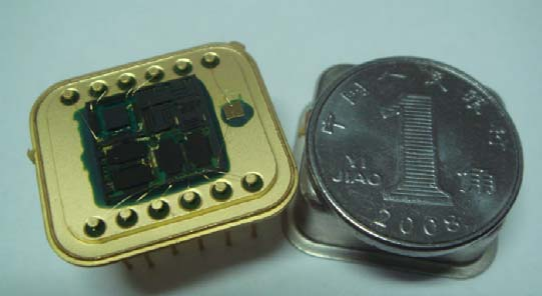
Photo of the final MIMU.

**Figure 14.**
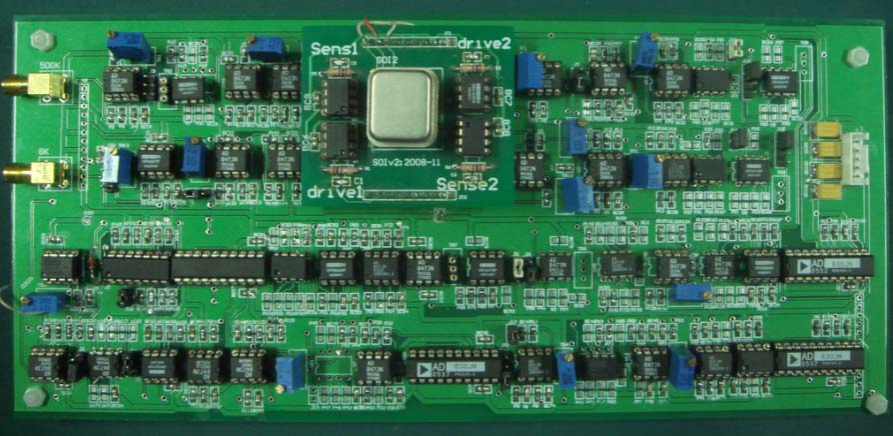
Photo of a test circuit board for MIMU.

We used the process flow shown in Figure 2 to fabricate the designed MIMU. The fabricated chip is shown in Figure 13. We used the circuit board shown in Figure 14 to test the MIMU.

**Figure 15.**
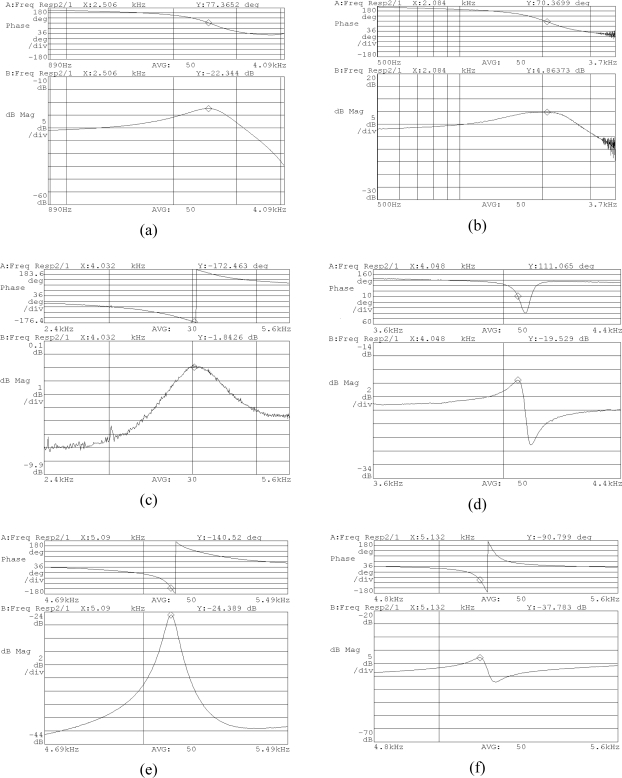
The frequency responses of the six inertial sensors. (a) X/Y-axis accelerometer; (b) Z-axis accelerometer; (c) X/Y-axis gyroscope in drive mode; (d) X/Y-axis gyroscope in sense mode; (e) Z-axis gyroscope in drive mode; (f) Z-axis gyroscope in sense mode.

The frequency responses of the six sensors are shown in Figure 15. It can be observed that the resonant frequencies of the X/Y-axis accelerometer and Z-axis accelerometer are about 2,506 Hz and 2,084 Hz, respectively. The resonant frequencies of the drive mode and sense mode for the X/Y-axis gyroscope are 4,032 Hz and 4,048 Hz, respectively. The resonant frequencies of the drive mode and sense mode for the Z-axis gyroscope are 5,090 Hz and 5,132 Hz, respectively.

**Figure 16.**
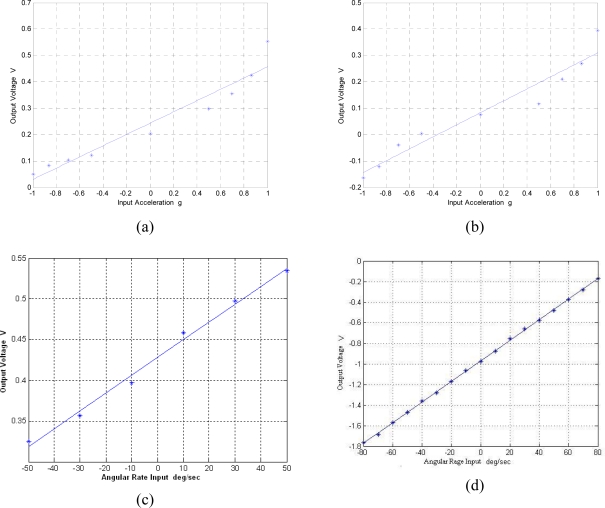
Scale factors for the six sensors in the MIMU. (a) X/Y-axis accelerometer; (b) Z-axis accelerometer; (c) X/Y-axis gyroscope; (d) Z-axis gyroscope.

The scale factor plots of the six sensors are shown in Figure 16. It can be seen that the scale factors of the X/Y-axis accelerometer and Z-axis accelerometer are about 213.3 mV/g and 226.9 mV/g, respectively. The big nonlinearity exists just because the rough test method based on a triangle ruler. The accelerometers were tiled 0°, 30°, 45°, 60°, 90°, 270°, 300°, 315°, 330°, 360°, respectively, through a triangle ruler to generate multiple acceleration input. The scale factors of the X/Y-axis gyroscope and Z-axis gyroscope are about 2.2 mV/°/s and 10.8 mV/°/s, respectively.

**Figure 17.**
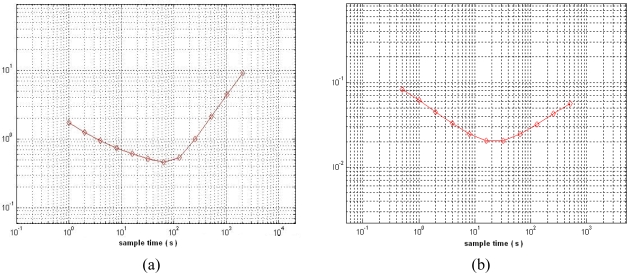
Plots of Allan variance for gyroscopes. (a) X/Y-axis gyroscope; (b) Z-axis gyroscope.

Plots of the Allan variance analysis for the gyroscopes are shown in Figure 17. It can be seen that the bias stability of the X/Y-axis gyroscope and the Z-axis gyroscope are about 2,135 deg/h and 80 deg/h, respectively.

**Figure 18.**
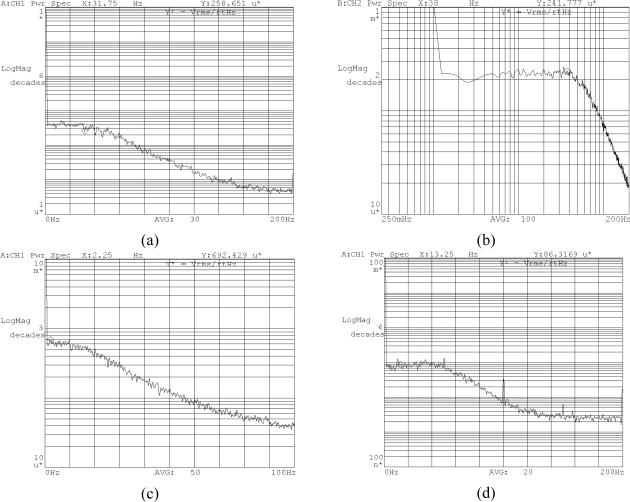
The noise floors of the six sensors in the MIMU. (a) X/Y-axis accelerometer; (b) Z-axis accelerometer; (c) X/Y-axis gyroscope; (d) Z-axis gyroscope.

The noise floors of the six sensors are shown in Figure 18. It can be observed that the power spectral densities of the noise for X/Y-axis accelerometers, Z-axis accelerometers, X/Y-axis gyroscopes, and Z-axis gyroscopes are $258.7\;\mu V/\sqrt{\text{Hz}}$, $247.8\;\mu V/\sqrt{\text{Hz}}$, $692.4\;\mu V/\sqrt{\text{Hz}}$, and $86.3\;\mu V/\sqrt{\text{Hz}}$, respectively. Consequently the resolutions of X/Y-axis accelerometers, Z-axis accelerometers, X/Y-axis gyroscopes, and Z-axis gyroscopes are $0.0012\;g/\sqrt{\text{Hz}}$, $0.0011\;g/\sqrt{\text{Hz}}$, $0.314\;{}^{\circ}/s/\sqrt{\text{Hz}}$, and $0.008\;{}^{\circ}/s/\sqrt{\text{Hz}}$, respectively.

It is obvious that the performance is far from the navigation level [38]. The major reason for this low performance lies in the capacitive detection circuit. There are a significant amount of noise that reduces the capacitance detection capability far below the expected 10^−19^ *F*. If the interface circuit were improved, the performance of the sensors would be much better. Another phenomenon is that the performance of the X/Y-axis gyroscope is much worse than that of the Z-axis gyroscope, primarily because the big squeeze film damping for the out-of-plane motion in the sense mode of the X/Y-axis gyroscope is packaged at one atmosphere. The large damping greatly reduced the quality of the sense mode. In the future, the X/Y-axis gyroscope needs to be improved further to obtain a better performance, e.g., by using a larger layout area or a smaller resonant frequency, if identical performance is required for all three gyroscopes.

## 6. Conclusions

In this paper, we demonstrated a miniature bulk micromachined MIMU; a detailed design, the fabrication process and testing were all discussed for the first time. The proposed global area optimization approach is proved to be very effective to determine the layout configuration of the six single-axis sensors in the mechanical element chip. Moreover, the widely used common geometry patterns are also proved to effectively alleviate the RIE lag effect in bulk micromachining process. The test results show that the current MIMU achieves medium accuracy. With an improved interface circuit, this technology can yield better performance than the surface micromachined MIMU.

## Figures and Tables

**Table 1.** The common geometry configuration for MIMU.

**Parameters**	**Values**
The overlap of comb fingers	80 μm
Length of comb fingers	160 μm
Width of comb fingers	4 μm
Gap between comb fingers	4 μm
Gap between the structure and substrate	3 μm
Thickness of structure	65 μm
Length of torsional beams	≤300 μm
Width of torsional beams	≥20 μm
Length of bending beams	≤550 μm
Width of bending beams	≥10 μm

**Table 2.** The side length of the equivalent square for sensors in the MIMU.

**Name of Sensors**	**Side Length**	**Notes**
X/Y-axis Accelerometer	$L_{\mathrm{ax}}=\sqrt{2\times n\times l_{\mathrm{cay}}\times d_{\mathrm{cay}}\times 4\times 4}$	*l_cay_* =80*μm*; *d_cay_*=4*μm*
Z-axis Accelerometer	$L_{\mathrm{az}}=\sqrt{2w_{\mathrm{maz}}\times l_{\mathrm{maz}}}$	*l_maz_* = *w_maz_*
X/Y-axis Gyroscope	$L_{\mathrm{gy}}=\sqrt{2\times \left(w_{\mathrm{mgy}}+l_{\mathrm{cgy}}\right)\left(l_{\mathrm{mgy}}+2l_{\mathrm{bgy}}\right)}$	*w_mgy_* = *l_mgy_*; *l_cgy_* =500*μm*; *l_bgy_* =550
Z-axis Gyroscope	*L_gz_* = *l_mgz_* + 2 × (*l_bgz_* − 50)	*l_bgz_* =550

**Table 3.** The common parameters for MIMU optimization.

**Parameters**	**Values**
The material density of the silicon	2.33 × 10^3^ kg/m^3^
Young’s modulus	130 Gpa
Poisson’s ratio	0.22
Shear modulus	53.3 GPa
Packaging pressure	1 atm
Area limit for MIMU	10 mm × 10 mm

**Table 4.** Dimensions of X/Y-axis accelerometer.

*l_may_*	2,000 μm	*d_cay_*	4 μm
*w_may_*	780 μm	*l_cay_*	80 μm
Folded beams	Consists of nine beams, each with a length of 360 μm.		

**Table 5.** Dimensions of Z-axis accelerometer.

*l_baz_*	170 μm	*w_maz_*	1,200 μm
*w_baz_*	20 μm	*l_haz_*	1,300 μm
*w_haz_*	540 μm	*l_maz_*	2,000 μm

**Table 6.** Dimensions of X/Y-axis gyroscope.

*l_bgy_*	530 μm	*w_tgy_*	20 μm
*w_bgy_*	10 μm	*l_mgy_*	2,000 μm
*l_cgy_*	500 μm	*w_mgy_*	1,500 μm
*l_tgy_*	200 μm	*d_cgy_*	4 μm

**Table 7.** Dimensions of Z-axis gyroscope.

*l_bgz_*	520 μm	*l_mgz_*	2,000 μm
*w_bgz_*	10 μm	*l_cgz_*	80 μm
*d_cgz_*	4 μm	*l_cgz_*	160 μm

**Table 8.** Comparison of the equivalent side length with parameters adjusted.

	**Global Layout Configuration**	**Final Layout Configuration**
X/Y-axis Accelerometer	1,500 μm	1,452 μm
Z-axis Accelerometer	1,500 μm	1,697 μm
X/Y-axis Gyroscope	3,000 μm	3,498 μm
Z-axis Gyroscope	3,000 μm	3,000 μm
